# A B C of the Swedish System of Educational Gymnastics

**Published:** 1892-06

**Authors:** 


*A B C of the Swedish System of Educational Gymnastics.* By HARTIVG NISSEN. With 77 Illustrations. Pp. 107. Philadelphia: F. A. DAVIS. 1891.

Herein is contained a description of the regular series of gymnastic exercises of the Swedish system. The author has had a large practical experience, and has compiled this book for the use of teachers in schools. The well-known advantage of the Swedish system lies in its affording a complete course of muscular exercises for which the use of appliances is unnecessary. Directions are given for the carrying out of graduated exercises, arranged in progressive series from the simple to the complex. These directions are given in the briefest possible terms. This, although in accordance with the practical character of the book, is apt to lead to obscurity in the wording. Generally speaking, the directions are clear; but in some instances it is difficult to make out their precise meaning without prolonged study. We also doubt the exactness of the physiological explanations which are given of the object of some of the exercises. The illustrations are numerous and an aid to the reader. No doubt the book will be found useful by those for whom it has been written.

